# Immune-Stimulatory Effects of *Althaea rosea* Flower Extracts through the MAPK Signaling Pathway in RAW264.7 Cells

**DOI:** 10.3390/molecules22050679

**Published:** 2017-04-25

**Authors:** Yon-Suk Kim, Eun-Kyung Kim, Weligala Pahalagedara Amila Srilal Nawarathna, Xin Dong, Woen-Bin Shin, Jin-Su Park, Sang-Ho Moon, Pyo-Jam Park

**Affiliations:** 1 Department of Biotechnology, Konkuk University, Chungju, Chungbuk 27478, Korea; kimyonsuk@kku.ac.kr (Y.-S.K.); amilasrilal@gmail.com (W.P.A.S.N.); 0527dongxin@gmail.com (X.D.); shinwoenbin@naver.com (W.-B.S.); pdjmh043@hanmail.net (J.-S.P.); 2 Nokyong Research Center, Konkuk University, Chungju, Chungbuk 27478, Korea; moon0204@kku.ac.kr; 3 Division of Food and Bio Science, Konkuk University, Chungju 27478, Korea; eunkyungkim@kku.ac.kr

**Keywords:** *Althaea rosea*, RAW264.7 cells, immune-stimulatory effect

## Abstract

*Althaea rosea* (Linn.) is a medicinal plant from China and Korea that has been traditionally used to control inflammation, to stop bedwetting and as a mouthwash in cases of bleeding gums. Its flowers are employed medicinally for their emollient, demulcent and diuretic properties, which make them useful in chest complaints. Furthermore, a flower extract decoction is used to improve blood circulation, for the treatment of constipation, dysmenorrhoea, haemorrhages, etc. However, the possible mechanisms of the immune-stimulatory effect remains to be elucidated. Therefore, we investigated the role of *Althaea rosea* flower (ARF) extracts in the immune-stimulatory effect of macrophages and the underlying mechanisms of action. ARF water extract (ARFW) could dose-dependently increase NO production and cytokines (IL-6 and TNF-α). We also found that ARFW significantly increased the expression of iNOS and COX-2 proteins in RAW264.7 cells. Consistent with these results, MAPK protein (JNK, ERK, p38) expression levels were induced after treatment with ARFW. Additionally, ARFW showed a marked increase in the phosphorylation level of IκBα and subsequent IκBα degradation allowing NF-κB nuclear translocation. These results suggest that the immune-stimulatory effect of *A. rosea* flower extracts is mediated through the translocation of NF-κB p65 subunit into the nucleus from the cytoplasm and subsequent activation of pro-inflammatory cytokines (IL-6 and TNF-α) and other mediators (iNOS and COX-2), which occurs mainly through MAPK signalling pathway. Thus, we suggest that ARFW could be considered as a potential therapeutic agent useful in the development of immune-stimulatory compounds.

## 1. Introduction

*Althaea rosea* (Linn.) (genus *Althaea*, family Malvaceae), commonly known as hollyhock or marshmallow is a perennial ornamental plant, which commonly grows in gardens, parks, riverbanks and salt marshes. This plant is native to China and is nowadays distributed over many parts over the world in tropical and temperate regions such as the Middle, Near East, Mediterranean and central Asian regions and southern Europe [1]. Chinese Traditional Medicine has been treating many kinds of diseases for a long time, and *Althaea rosea* is one of the traditional Chinese medicinal plants [2] The medicinal parts of *A. rosea* include its flowers, leaves, roots, and seeds [3]. *A. rosea* flowers and roots have long been used in traditional Uyghur medicine for the treatment of several diseases; this plant is used as an expectorant, coolant, diuretic, anti-inflammatory, febrifuge, demulcent, and astringent agent [4]. The seeds of *A. rosea* are regularly used by Uyghur physicians for the treatment of kidney and uterus inflammation [5] and flowers are regarded as bleeding stopping, swelling reducing and detoxifying in traditional Uyghur medicine [2].

An analysis of the flavonoid fraction from the hollyhock extract revealed the presence of quercetin and kaempferol [6]. Dudek et al. [7] investigated the phenolic acid content of *A. rosea* flowers using methanolic and methanolic-aqueous extracts of whole flowers, petals and calyxes. Cinnamic (ferulic, *p*-coumaric, caffeic), benzoic (*p*-hydroxybenzoic, vanillic, syringic), *p*-hydroxy-phenylacetic, *p*-coumaric, syringic and *p*-hydroxybenzoic acids were detected in almost all fractions obtained from different parts of the flowers. Five flavonoid compounds were isolated from the aerial parts of *A. rosea* and identified as quercetin 3-*O*-β-d-glucuronopyranoside-8-*C*-β-d-glucopyranoside, kaempherol-3-*O*-β-d-rutinoside, kaempherol-4′-*O*-β-d-glucoside, kaempherol-3-*O*-β-d-glucoside, and kaempherol [8]. Although this plant’s medicinal properties have been recognised for a long time before, especially in Chinese Traditional Medicine, only a few studies were devoted to the research of evaluating mechanisms of actions behind those different medicinal attributes of *A. rosea* flower.

Immuno-stimulants are generally classified as compounds which can enhance the non-specific immune system responses by activating the activities of phagocytes such as neutrophils and macrophages. Usually, most immune-stimulants activate the function of macrophages after specific binding with cell surface receptors. Toll-like receptor 4 (TLR4) is a type one transmembrane glycoprotein having the molecular weight of 100 kDa and it is found to be expressed in macrophages and other cells [9]. In recently years, many studies have revealed that the variety of plant extracts have the capacity to activate the toll-like receptor 4 (TLR4) signalling pathways. A number of downstream signaling pathways are triggered by different adaptor complexes interacting with TLR4, including NF-kB and mitogen-activated protein kinases (MAPKs), eventually leading to the generation of reactive oxygen species (ROS), nitric oxide (NO), as well as inflammatory cytokines/chemokines [10].

Macrophages are multifunctional cells and play important roles in host defence system. After activation of macrophage, they produce various inflammatory mediators and cytokines such as NO and TNF-α [11].

The focus of the present investigation was to explore whether *A. rosea* flower extracts could induce an immune inflammatory response. First, we assessed immune stimulatory capacities of *A. rosea* flower extracts by quantifying NO release and cytokine levels (IL-6, TNF-α). Furthermore, in order to elucidate the molecular mechanisms behind this effect, downstream inflammatory MAPK signalling pathway and phosphorylation of IkBα were examined. Here, we hypothesised that *A. rosea* flower extracts possess potent immune-stimulating properties by activating MAPK/NF-κB signaling pathway in macrophages.

In this study, we demonstrated that ARF water extract (ARFW) was involved in modulating the immune response of macrophage via MAPK pathway. Here we report that ARFW induces the expression of proteins in MAPK (JNK, ERK, p38) signalling pathway, IκBα degradation and subsequent increase in the production of pro-inflammatory cytokines (IL-6, TNF-α) and mediators (iNOS and COX-2) in RAW264.7 cells. The results suggest that *A. rosea* flower water extract could exert immuno-stimulatory effects through the activation of MAPK/ NF-κB signalling pathway.

## 2. Results

### 2.1. Effect of ARF Extracts on Cell Viability and NO Production in RAW264.7 Cells

**Figure 1:**
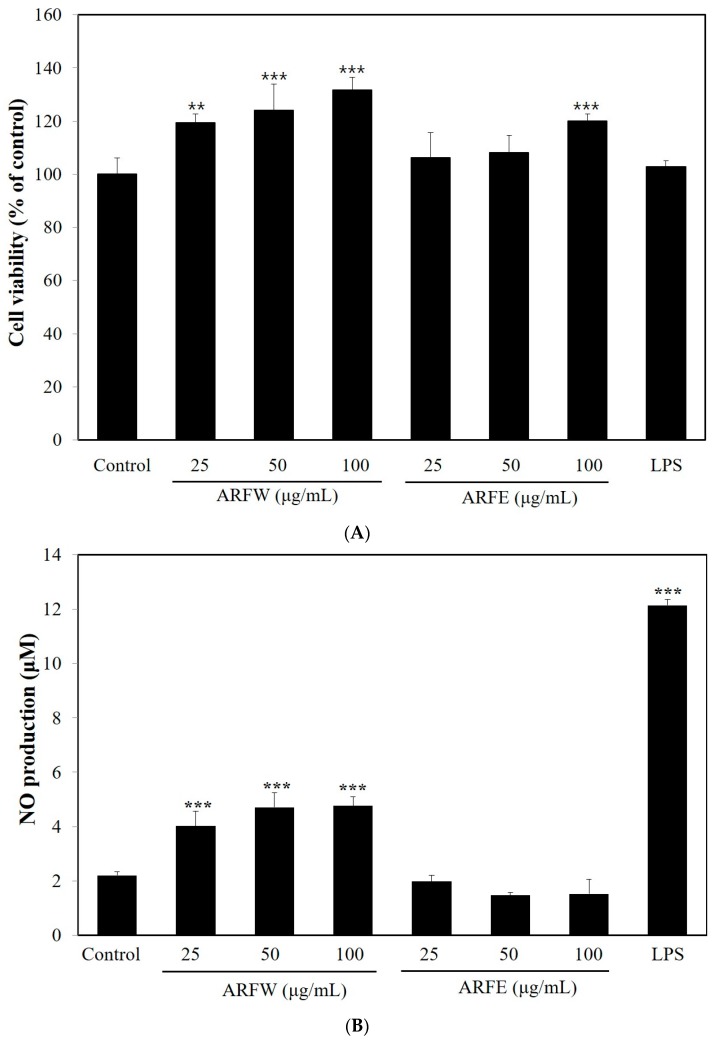
*A. rosea* flower extracts were not toxic to murine macrophage RAW264.7 cells (**A**) *A. rosea* flower water extracts (ARFW) induced the production of NO; (**B**) The cell viability was determined by MTT assay. ARFW, ARFE and lipopolysaccharide (LPS, 100 ng/mL) showed no cytotoxicity on RAW264.7 cells with 100 μg/mL maximum concentration for 24 h. NO production was induced by ARFW, but not by ARFE. ** *p* < 0.01, *** *p* < 0.001 vs. control.

First of all, we examined the effect of ARF extracts on the viability of RAW264.7 cells using MTT assay. As shown in Figure 1A, no cytotoxic effect was observed, when the cells were exposed to ARFW or ARF ethanol extract (ARFE) with 100 μg/mL maximum concentration for 24 h. Secondly, modulation of NO production in murine macrophage RAW264.7 cells by ARF extracts was used to figure out their immune-stimulatory effects. LPS was used as positive control & it markedly enhanced NO production in RAW264.7 cells. Treatment with *A. rosea* flower water extract (ARFW) significantly increased NO production, but there was no significant increase in NO production after treatment with *A. rosea* flower ethanol extract (ARFE) (Figure 1B). Thus, only ARFW was subjected to further analysis to explore its immuno-stimulating effect and mechanisms.

### 2.2. Effect of ARFW on the Expression of TNF-α and IL-6

**Figure 2:**
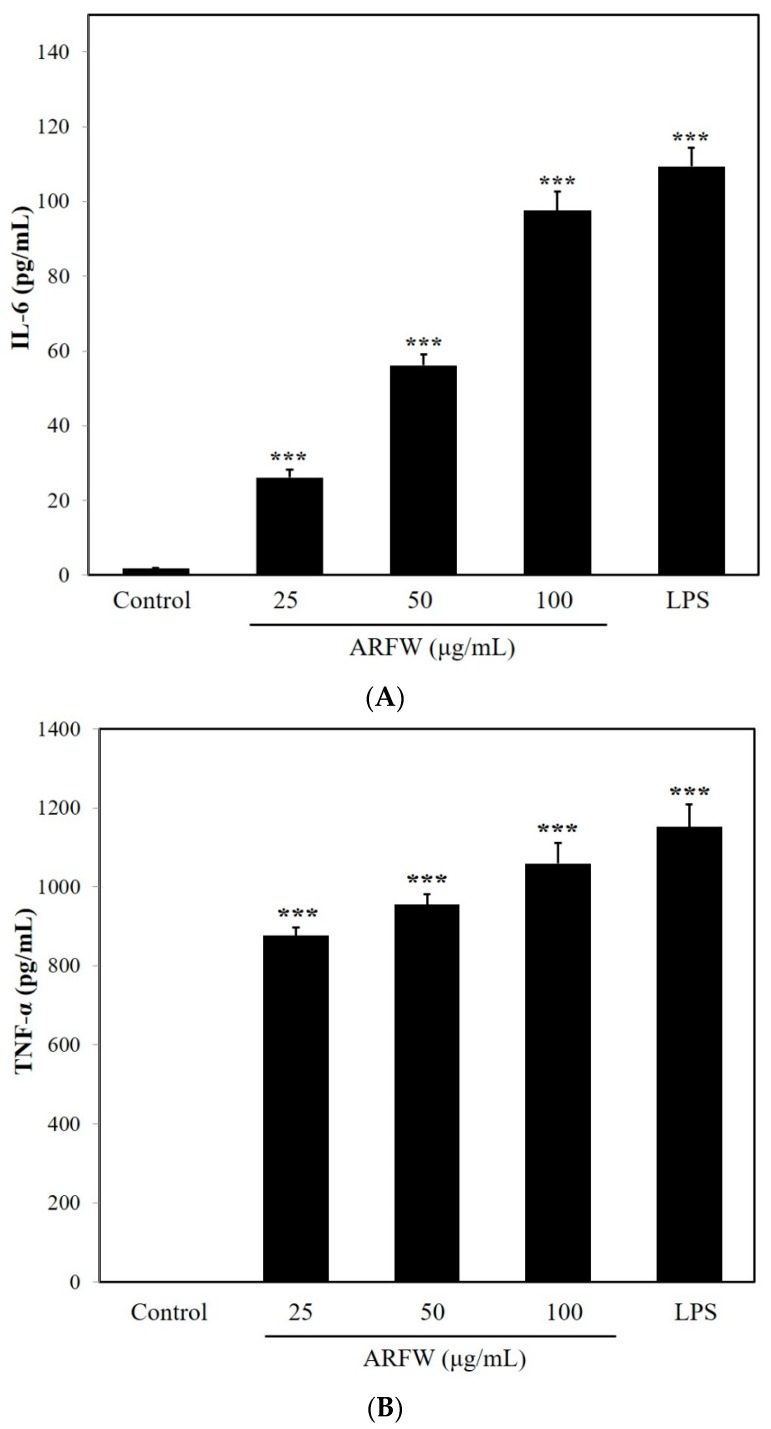
ARFW increased the release of cytokines (IL-6 (**A**); TNF-α (**B**)). The production of cytokines was determined by ELISA assay. All values are expressed as the means ± S.D. *** *p* < 0.001 vs control.

The proinflammatory cytokines including TNF-α and IL-6 are known to be potent immunomodulators in activated macrophages. Therefore, we determined TNF-α and IL-6 secretion levels in murine macrophage RAW264.7 cells after ARFW treatments. As shown in Figure 2, exposure to ARFW significantly increased the production of TNF-α and IL-6 compared to the control.

Many reports have shown that LPS (lipopolysaccharide) is a high-affinity agonist for the TLR4 signaling pathway [12] and TLR4 signalling stimulates increased production of NO, ROS and cytokines [10]. Consistent with the previous reports, LPS treatment showed a stimulated production of cytokines leading to immunostimulatory effect in RAW264.7 cells. Since ARFW treatment also could induce the production of TNF-α and IL-6, ARFW might possess the capacity to promote immunostimulatory effect in RAW264.7 cells.

### 2.3. Effect of ARFW on the Expression of iNOS and COX-2

**Figure 3:**
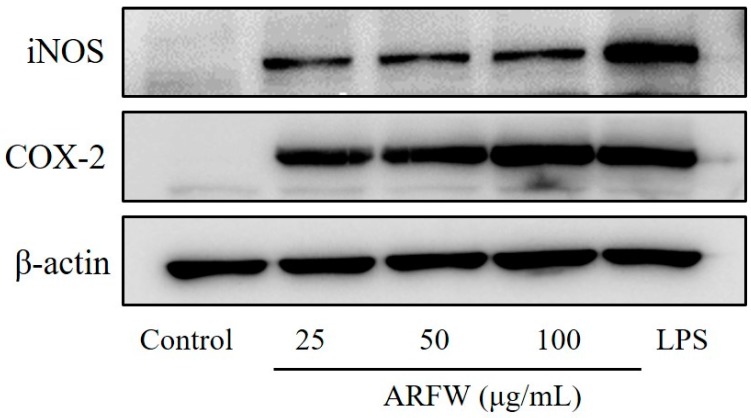
ARFW induced iNOS and COX-2 protein expression. Cells were treated with ARFW or LPS (100 ng/mL) for 18 h and protein expression levels were determined by western blot analysis with specific antibodies.

Inducible nitric oxide synthase (iNOS) is the enzyme, which is responsible for synthesising NO from L-arginine and expressed in a variety of cell types including macrophages. Cyclooxygenase COX-2 is associated with the conversion of arachidonic acid to prostaglandin E_2_ [13]. The iNOS and COX-2 protein expression levels in RAW264.7 cells were examined after ARFW treatments by western blot analysis to explore their effects on pro-inflammatory proteins. As shown in Figure 3, Treatments with different ARFW concentrations (25, 50, 100 μg/mL) for 18 h could markedly increase iNOS and COX-2 protein expression levels compared to control. Moreover, LPS treatment was performed as a positive control and LPS showed strong iNOS and COX-2 protein expression levels compared to control. The detection of β-actin was carried out in the same blot as an internal control. These results indicate that ARFW can upregulate the expression of pro-inflammatory mediator enzymes such as iNOS and COX-2.

### 2.4. Effect of ARFW on IkBa Degradation and NF-kB Activation

**Figure 4:**
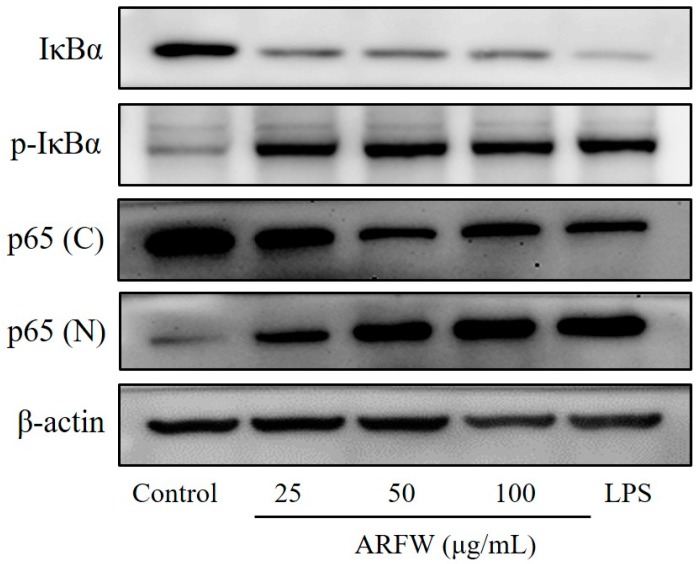
Effect of ARFW on NF-kB activity in RAW264.7 cells. Cells were treated with ARFW or LPS (100 ng/mL) for 15 min. Total cellular proteins were prepared and subjected to western blot analysis for determination of IκBα and *p*-IκBα protein levels. Nuclear (N) and cytosolic (C) proteins were isolated, and the levels of p65 were determined by Western blot analysis.

NF-κB is an important transcription factor to regulate pro-inflammatory mediators in stimulated macrophages. Activation of NF-κB mediates the transcription of numerous genes associated with stimulation of immune system, including pro-inflammatory enzymes iNOS and COX-2 [14]. To investigate whether ARFW affects the signalling pathways leading to NF-κB activation, RAW264.7 cells were treated with ARFW for 15 min, and then the cytoplasmic level of IκBα and nuclear level of NF-κB p65 subunit were determined using western blot analysis. As shown in Figure 4, treatment with ARFW increased phosphorylation levels of IκBα and NF-κB p65 subunit in the nucleus. Induced phosphorylation level of IκBα leads to its subsequent ubiquitination and degradation via the proteasome pathway leading to translocate freed NF-κB into the nucleus. Furthermore, Induced phosphorylation level of NF-κB p65 subunit together with nuclear translocation lead to increased NF-κB DNA-binding ability in RAW264.7 cells. These results indicated that ARFW could activate NF-κB leading to transcription of target genes including pro-inflammatory enzymes iNOS and COX-2 in macrophages.

### 2.5. Effect of ARFW on MAPK Signalling Activation

**Figure 5:**
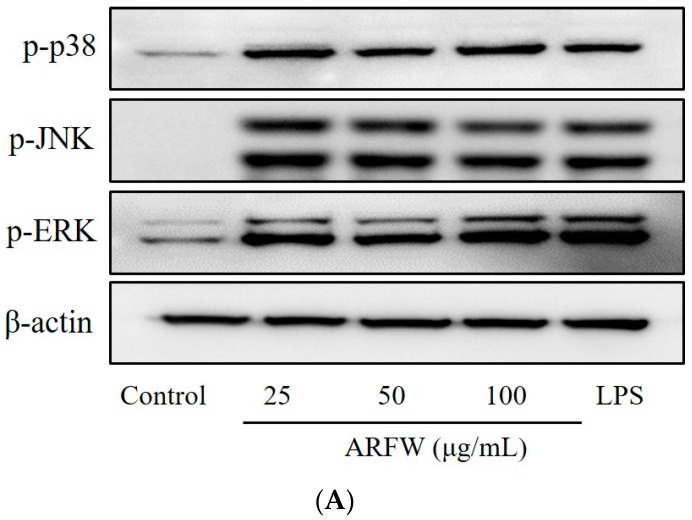

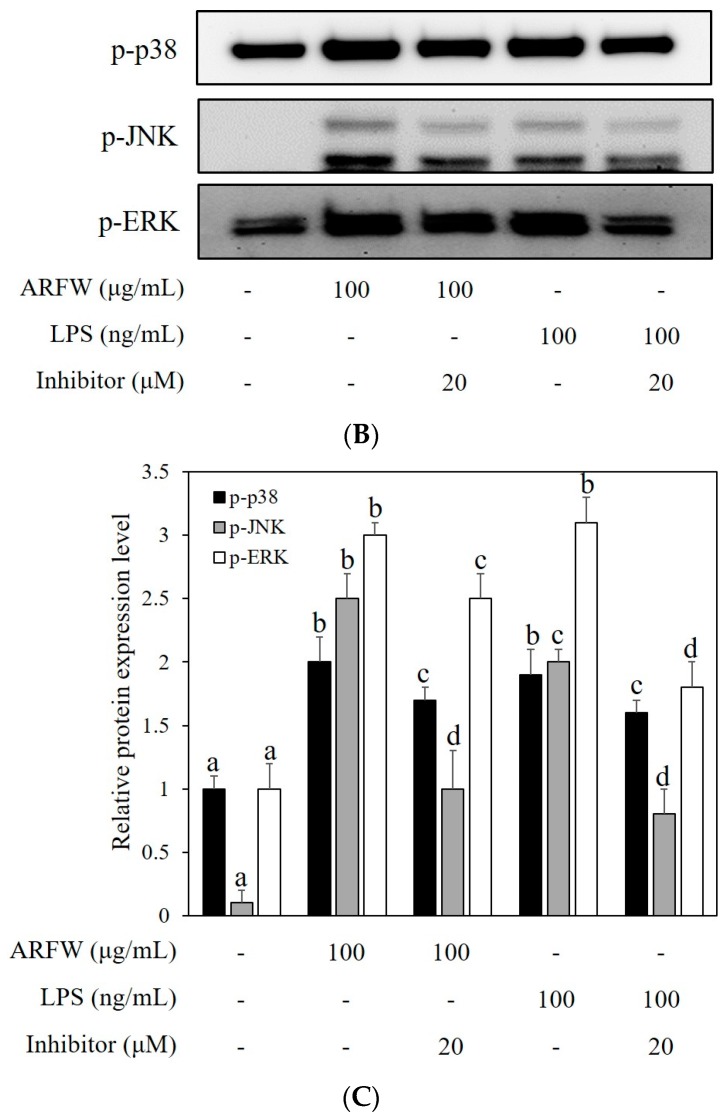
Effect of ARFW on MAPK signaling. Cells were treated with ARFW or LPS (100 ng/mL) for 30 min and MAPK protein expression levels were determined by western blot analysis with specific antibodies (**A**). Cells were treated with ARFW or LPS (100 ng/mL) in the presence of MAPK inhibitors for 30 min and MAPK protein expression levels were determined, Cells were treated with 20 μM of the MAPK inhibitor SB203580 (p38 inhibitor), SP600125 (JNK inhibitor), and PD098059 (ERK inhibitor) (**B**,**C**).

To investigate whether the increased production of iNOS and COX-2 by ARFW is mediated through MAPK pathway, next we evaluated the effect of ARFW on MAPK family proteins, p38, JNK, ERK1/2 by Western blot analysis. As shown in Figure 5A, we found that phosphorylation levels of MAPK isoforms, p38, JNK, ERK1/2 were markedly increased after 30 min treatment with ARFW or LPS (Positive control). To confirm whether ARFW is involved in the activation of MAPK isoforms, RAW264.7 cells were treated with ARFW (100 μg/mL) and LPS (100 ng/mL) in the presence of ERK inhibitor (PD098059), p38 inhibitor (SB203580) and JNK inhibitor (SP600125).

Treatment of ARFW or LPS markedly increased phosphorylation levels of p38, JNK, and ERK1/2, however, the presence of MAPK inhibitors with ARFW or LPS significantly inhibited phosphorylation of p38, JNK, ERK1/2 (Figure 5B,C). All these observations suggested that ARFW could stimulate the MAPK pathway, which might lead to increased production of iNOS and COX-2 through NF-κB activation.

## 3. Discussion

A well-functioning immune system is very critical for staying healthy. Thus, natural substances which can strengthen the immune system have been being explored for a long time. Many different synthetic preparations are claimed to be immune modulators, which could provide beneficial effects for fighting infections. However, many artificial immune-stimulators are found to have undesirable side effects. Hence, natural substances with immune-stimulatory benefits would be a promising alternative for those synthetic compounds. In the current study, we evaluated the immunomodulatory effect of ARF extracts using RAW264.7 cells.

Many studies have demonstrated that polysaccharides isolated from medicinal plants or food increased macrophage cytotoxic activity against tumor cells and microorganisms. This effect is found to be mediated through activated phagocytic activity, increased reactive oxygen species (ROS) and nitric oxide (NO) production, and enhanced production of cytokines and chemokines, including tumor necrosis factor (TNF-ɑ), interleukin IL-1, IL-6, IL-8, IL-12 [15,16,17]. The murine macrophage RAW264.7 cell line has been used as an in vitro model to evaluate immunomodulatory effects by determining synthesis of inflammatory molecules to different stimuli [18]. Macrophages and neutrophils play important roles in the host defence mechanism against pathogens by producing reactive oxygen species (ROS), nitric oxide (NO) and cytokines in the human body [19]. NO has a beneficial role in anti-tumor and anti-virus replication, even though overproduction of NO is associated with inflammatory and autoimmune diseases [20]. Our results showed that ARFW extract has the capacity to increase NO production in RAW264.7 cells. The production of pro-inflammatory mediators such as NO and prostaglandin E_2_ (PGE_2_) are induced by the inducible nitric oxide synthase (iNOS) and cyclooxygenase-2 (COX-2), respectively [21]. Consistent with previous reports, ARFW induced the production of NO by upregulating the expression of iNOS and COX-2 protein levels in RAW264.6 cells. The treatments of ARFW on RAW264.6 cells markedly induced the production of the pro-inflammatory cytokines such as TNF-α, IL-6.

It is a very well established fact that NF-κB protein is a transcription factor involved in various cellular responses including stimulation of immune system through the activation of pro-inflammatory genes such as TNF-α, IL-6, iNOS and COX-2 [22]. Hence, we evaluated the effect of ARFW on the activation of NF-κB protein in RAW264.6 cells. Our results indicated that ARFW is associated with a marked increase in the activation of NF-κB protein by inducing the phosphorylation of its inhibitor IκBα leading to its subsequent ubiquitination and degradation via the proteasome pathway and subsequent translocation of freed NF-κB into the nucleus.

In addition, recent studies have shown that activation of MAPKs, such as ERK, JNK, and p38, is generally involved with the immune-stimulatory activity of macrophage [23]. The present study demonstrated that ARFW could activate the downstream MAPK signalling pathway by upregulating the phosphorylation levels of ERK, JNK, and p38 proteins in RAW264.7. Therefore, ARFW stimulated increased expression of ERK1/2, JNK and ERK and it was a strong inducer of cytokine expression in RAW 264.7 cells. MAPK signalling pathways, which include many proteins like the extracellular signal-regulated kinase1/2 (ERK1/2), p38, cMYC and c-Jun *N*-terminal kinase (JNK), regulate the activities of many transcription factors [24]. The activation of ERK is thought to be involved in macrophage activity, including increased production of pro-inflammatory cytokines. In addition, p38 MAPK activated by LPS has been postulated to play an important role in the control of TNF-α gene expression [25]. The JNK could be activated by environmental stress and some pro-inflammatory cytokines, also has an important role in immune system signalling [26,27].

## 4. Materials and Methods

### 4.1. Materials

Lipopolysaccharide (LPS), 3-(4,5-dimethylthiazol-2-yl)-2,5-diphenyltetrazolium bromide (MTT) and DCF-DA were purchased from Sigma Chemical Co. (St. Louis, MO, USA). *Althaea rosea* flowers were obtained from Hanbang Herbal Drug Co. (Jecheon, Korea). Bradford reagent was obtained from BioRad (Hercules, CA, USA); antibodies against NF-kB p65, iNOS, COX-2 were brought from Santa Cruz Biotechnology, Inc., (Santa Cruz, CA, USA); Antibodies against IκBα, p-IκBα, p-p38, p-JNK, p-ERK were purchased from Cell Signaling Technology (Beverly, MA, USA). All other reagents were of the highest grade commercially available.

### 4.2. Preparation of ARF Extracts

Aqueous and ethanol ARF extracts have been prepared as described below. Briefly, *Althaea rosea* dried flowers (100 g, ARF) were extracted with distilled water or 70% ethanol. The water extract was obtained by refluxing for 90 min at room temperature and ethanol extract was prepared by refluxing two times with 70% ethanol for 1 day at room temperature and then both fractions were filtered through filter paper (Whatman No. 41, GE Healthcare Life Sciences, Little Chalfont, Buckinghamshire, UK) under suction. The filtered extracts were evaporated under reduced pressure using a rotary vacuum evaporator (EYELA, Tokyo, Japan) at 50 °C. After evaporation, the water and ethanol extracts were lyophilized using a freeze dryer (Samwon Industry, Seoul, Korea).

### 4.3. Cell Culture

The murine macrophage RAW264.7 cells were purchased from the Korean Cell Line Bank (Seoul, Korea) and maintained in high glucose Dulbeco’s Modified Eagle’s Media (DMEM; Thermo Fisher Scientific, Waltham, MA, USA) supplemented with 10% heat-inactivated Fetal Bovine Serum (FBS), 100 U/mL penicillin and 100 μg/mL streptomycin at 37 °C humidified incubator having 5% CO_2_ and 95% air environment.

### 4.4. Cell Viability

The cytotoxic effects of samples on RAW264.7 cells were evaluated using 3-(4,5-dimethylthiazol-2-yl)-2,5-diphenyltetrazolium bromide (MTT) assay. Briefly, RAW264.7 cells were seeded in 96-well plates at a density of 2 × 10^4^ cells/well. After overnight growth, adhered cells were treated with different concentrations of water and ethanol ARF extracts (25–100 μg/mL) or LPS (100 ng/mL) for another 24 h. After 24 h incubation period, MTT solution was added and the cells were further incubated for 2 h. After that, the supernatant was removed and then the resulting formazan crystals were dissolved in DMSO. The absorbance at 540 nm was measured with a microplate reader (SpectraMax M2/M2e; Molecular Devices, Sunnyvale, CA, USA).

### 4.5. Measurement of Nitric Oxide (NO) Generation

The concentration of nitric oxide (NO) in the cell culture medium was analysed by determining the accumulation of nitrite (NO_2_^−^) in the cell culture medium by Griess reagent using sodium nitrite (NaNO_2_) as the standard. The RAW264.7 cells were treated with ARF extracts for 18 h, and then the culture medium (100 μL) was transferred into each well of 96-well plate. The equal volume (100 μL) of Griess reagent was also added to each well and incubated for 10 min at room temperature. The absorbance at 550 nm was measured with a microplate reader (SpectraMax M2/M2e; Molecular Devices, Sunnyvale, CA, USA).

### 4.6. Determination of Inflammatory Cytokine Production

RAW264.7 cells (1 × 10^5^ cells/well) were seeded into 24-well plates and incubated overnight for cell adherence. After treatment with water extract (ARFW) or LPS (as a positive control) for 18 h, the release of TNF-α and IL-6 in the cell supernatants were assayed using ELISA kits (R&D Systems, Minneapolis, MN, USA) according to the manufacturer's instructions.

### 4.7. Western Blot Analysis

After sample treatments, the treated cells were harvested, lysed and protein concentration was quantified by Bradford assay. Samples containing (20–30 μg) proteins were subjected to sodium dodecyl sulfate-polyacrylamide gel electrophoresis (SDS-PAGE; BioRad Laboratories, Hercules, CA, USA) and separated proteins were transferred to polyvinylidene fluoride (PVDF) membranes (GE Healthcare, Buckinghamshire, UK). After blocking with 5% skim milk in Tris-buffered saline-Tween 20 solution (TBST) for 1 h at room temperature, the membranes were incubated overnight with specific primary antibodies (dilution 1:1000) at 4 °C. After three times washing with TBST, the membranes were incubated with species appropriate HRP-conjugated secondary antibodies (dilution 1:5000) for 1 h at room temperature. Protein bands were developed using an enhanced chemiluminescence (ECL) reagent and the immune blots were visualised using Davinch-Chemi™ imaging system (Young Wha Scientific Co. Ltd., Seoul, Korea) and densitometry data were normalised to the β-actin.

### 4.8. Statistical Analysis

All of the experiments were carried out with triplicates. Values are shown as means ± standard deviation (SD) and they were analysed by analysis of variance (ANOVA), followed by Dunnett’s test to determine the significance of differences using GraphPad Prism 5 (Graph Pad Software Inc., San Diego, CA, USA), *p* < 0.05 was considered statistically significant.

## 5. Conclusions

In summary, we have demonstrated that ARFW exerts immune-stimulatory effects on murine macrophage RAW264.6 cells through the activation of MAPK/NF-κB downstream signalling pathway leading to increased expression of pro-inflammatory cytokines and mediators. Further studies are needed to find out the main compound, which is responsible for ARFW’s immune stimulatory effect.
